# The cross-level influence of ethical leadership on employee’s OCBE: a two-wave study based on the social identity approach

**DOI:** 10.3389/fpsyg.2023.1270359

**Published:** 2023-11-30

**Authors:** Xiaoyan Su, Haipeng Wang, Yuyu Zhu

**Affiliations:** International Business School, Jinan University, Zhuhai, China

**Keywords:** ethical leadership, organizational citizenship behavior for the environment, team environmental atmosphere, leader identity, cross-level influence

## Abstract

The importance of organizational citizenship behavior for the environment (OCBE) has received increasing attention in recent years because organizations face increasing pressure from environmental deterioration. The purpose of this study is to use social identity theory to construct a cross-level theoretical model of ethical leadership on OCBE, and to explore the cross-level influential mechanisms of ethical leadership on OCBE. Data collection was conducted via a two-wave distribution of leader-employee paired questionnaires in 20 manufacturing companies in China. In the first wave, data about OCBE and team environmental atmosphere were collected from leaders. Subsequently, 2 months later, we conducted the second wave of data collection when data about ethical leadership and leader identity were obtained from their employees. The results showed that at the individual level, ethical leadership has a significant positive impact on employees’ OCBE, and such relationship is partially mediated by employees’ leader identity and positively moderated by team environmental atmosphere across levels. At the team level, ethical leadership has a significant positive impact on employees’ OCBE, and such relationship is completely mediated by team environmental atmosphere. This study investigates the cross-level influential mechanism of ethical leadership on OCBE in China and provides theoretical guidance for enterprises to promote OCBE effectively.

## 1 Introduction

In recent years, with the rapid development of China’s economy, the problem of resource depletion and environmental pollution has become increasingly prominent, and the call for protecting the ecological environment and achieving the sustainable development of society, economy and environment has become increasingly high. The data show that China’s Environmental Performance Index ranking has dropped from 94th in 2006 to 160th in 2022 (Wolf et al., 2022), and the ecological and environmental situation was not optimistic. In response to the ecological and environmental problem, the Chinese government proposed to build a resource-saving and environment-friendly society as early as the 12th Five-Year Plan. The Chinese government made a solemn commitment to achieve its carbon peak by 2030 and accomplish its carbon neutrality goal by 2060 at the General Assembly of the United Nations. The report to the 20th National Congress of Communist Party of China proposed the promotion of green development. In this context, China’s Environmental Protection Law, Air Pollution Control Law, Garbage Sorting Regulations, and other regulations have been implemented gradually. As the ecological environment deteriorates, enterprises have gained a profound understanding of how to balance economic interests and environmental protection, and have gradually accepted the necessity of sustainable development and green manufacturing (Bansal and Song, 2017).

The effectiveness of companies’ environmental protection relies not only on hard regulations of the government but also on the active response from employees, and companies have to promote employee engagement in green behaviors if they want to become a “sustaining organization” (Benn et al., 2014, p. 3). However, existing literature mainly focused on corporate strategy and operations, such as green innovation strategy (Huang and Li, 2017), environmental strategy, and green supply chain strategy (Klassen and McLaughlin, 1996), hence, there are relatively few studies that analyze employees’ green behavior at the micro-individual level (Galpin and Whittington, 2012; Robertson and Carleton, 2018). Green behavior refers to a series of pro-environmental behaviors, such as resource conservation, energy consumption reduction, and ecological protection (Afsar and Umrani, 2020). In fact, active employee participation in pro-environmental activities has an essential influence on the sustainable development of enterprises. Therefore, studying the topic of organization environmental protection from the micro-level of employees’ behavior is highly necessary.

Organizational citizenship behavior for the environment (OCBE) is a form of pro-environmental organizational citizenship behavior that is discretionary and performed by employees beyond their job requirements to achieve corporate environmental goals (Daily et al., 2009; Norton et al., 2015). Employees’ OCBE has received increasing attention because it can meet the environmental protection demands of stakeholders and promote the sustainable development of the organization (Ullah et al., 2021). Existing literature on the antecedents of employees’ OCBE has mainly focused on organizational factors, such as gren human resource management (GHRM) (Liu et al., 2020), environmental management system (EMS) (Khan et al., 2021), green absorptive capacity (Sarmad et al., 2023), organizational support (Lamm et al., 2013), and individual factors, such as environmental knowledge (Ahmed et al., 2020), CSR perceptions (Wells et al., 2015; Cheema et al., 2019; Tian and Robertson, 2019). It has been suggested that the mechanisms of how leadership styles influence employees’ OCBE should be the focus of subsequent research (Temminck et al., 2015). Current studies have explored the influence of responsible leadership (Afsar et al., 2016; Afsar and Umrani, 2020), transformational leadership (Kura, 2016; Crucke et al., 2022; Liu and Yu, 2023), spiritual leadership (Afsar et al., 2018; Robertson and Carleton, 2018), and ethical leadership (Avey et al., 2011; Lu and Lin, 2014) on employees’ green behaviors. Employees’ OCBE is inherently ethical, and the generation of such behavior is likely to be closely related to leaders’ ethical behavior (Organ et al., 2006). The ancient Confucian culture in China advocated for the unity of man and nature, promoting harmonious coexistence between humans and the natural environment. Ethical leadership is rooted in traditional Chinese Confucian culture, which places emphasis not only on individual adherence to moral norms, but also on the ability to guide and educate subordinates through moral leadership, exerting an implicit influence on their behavior. Ethical leaders place special emphasis on building relationships with various stakeholders. These may include relationships between organizations and society, organizations and the natural environment, organizations and other organizations, and organizations and individuals; among these, the relationship between organizations and the natural environment is of great significance; however, there exists a dearth of studies delving into the influence of ethical leadership on employees’ pro-environmental behavior (Ahmad et al., 2021).

According to the social identity theory (Tajfel and Turner, 1986), frequent interaction and communication between employees and leaders can deeply influence employees with the leader’s environmental values. Employees define themselves in accordance with such values, and hence their interests are closely connected with leader’s interests, generating their identity with the leader’s values. Employees’ identity with their leader can effectively promote the cognitive and behavioral changes among subordinates, and encourage them to align with the leader (Hogg, 2001; Shao et al., 2008; Hogg et al., 2012). At the same time, ethical leadership, as an important source of information in organizations, affects employees’ perceptions of team context, and the resulting team environmental atmosphere can also have an impact on employees’ OCBE. Therefore, this study will further explore the relationship between ethical leadership and employees’ OCBE and analyze the mechanisms and boundary conditions of this relationship. In addition, existing literature on ethical leadership was mainly limited to individual level, less on team level, and even less on both individual and team levels. Norton et al. (2015) have suggested that a multilevel theoretical model should be adopted for analyzing ethical leadership in further studies. This implies that ethical leadership, as a multilevel oriented leadership style, requires a comprehensive examination of its influence from both individual and team levels. Therefore, this study aims to use social identity theory to construct a cross-level theoretical model of ethical leadership on employees’ OCBE and to explore the cross-level influential mechanisms of ethical leadership on employees’ OCBE.

The structure of this paper is as follows: in the second section, we delineate the theoretical linkage between ethical leadership, leader identity, team environmental atmosphere and employees’ OCBE across levels; in the third section, we discuss our research methods, including data collection method, variable measurement, and statistical method; in the fourth section, we conduct empirical analysis to test research hypotheses and interpret the results in a detailed manner; in the end, we put forward research conclusions with a discussion about the theoretical and managerial implications of the research findings.

## 2 Literature review and hypotheses development

### 2.1 Theoretical basis

The concept of ethical leadership was first introduced by Enderle and is considered as a leadership style that emphasizes moral standards and ethical management (Enderle, 1987). Ethical leadership can be defined as “the demonstration of normatively appropriate behavior in both personal and interpersonal contexts and the active promotion of socially responsible behavior at all levels in the organization reinforcing a moral ethos through communication and ethical decision making” (Tourigny et al., 2019, p. 429). This implies ethical leadership embodies both a moral person and a moral manager (Shareef and Atan, 2019). As a moral person, an ethical leader possesses and exhibits personal qualities that align with ethical principles such as honesty and integrity, and is capable of making ethical decisions (Trevino et al., 2000; Wang et al., 2021). As a moral manager, an ethical leader actively implements ethical management practices and conducts ethical behaviors that benefit the interests of various stakeholders, including employees, organizations, and society (Mayer et al., 2012; Islam et al., 2021a). For example, ethical leaders establish ethical and moral frameworks within their organization, transfer moral philosophies to employees through two-way communication, and encourage employees to emulate similar ethical behavior (Islam et al., 2021b). Trevino et al. (2000) argued that ethical leaders have the ability to influence their subordinates’ thoughts and behaviors and play a pivotal role in developing corporate ethics. Based on existing literature, this study divides ethical leadership into individual-level ethical leadership and team-level ethical leadership. Individual-level ethical leadership pertains to how individual employees evaluate the ethical behavior of their leaders, while team-level ethical leadership refers to the collective perception of ethical leadership styles among team members (Liao and Chuang, 2007).

Tajfel and Turner’s (1986) social identity theory argues that employees’ internalized identity with the leader and organization is the prerequisite for effective leadership. Employees will accept organizational standards and learn from the leader’s ethical norms when they generate identity with the leader (Brown et al., 2006). In light of social identity theory, ethical leaders can lead employees to identify with them by satisfying their ethical needs, transform their environmental values and norms to employees, and finally inspire employees to demonstrate more OCBE. Thus, leader identity can serve as a mediator that connects the relationship between individual-level ethical leadership and employees’ OCBE. In addition, ethical leaders can also influence subordinates to engage in OCBE by demonstrating similar behaviors and creating an environmental climate. Therefore, team environmental atmosphere can also serve as a cross-level mediator that bridges between team-level ethical leadership and employees’ OCBE. Stronger team environmental atmosphere can make employees more committed to the team environmental norms, which will strengthen the cross-level influence of ethical leadership on employees’ OCBE. Based on this, team environmental atmosphere can further serve as a contextual moderator for the relationship between individual-level ethical leadership and employees’ OCBE.

### 2.2 Individual-level ethical leadership and employees’ OCBE

Ethical leaders, actively aware of the organization-environment relationship, persistently apply environmental protection philosophies, establish organizational environmental protection frameworks, and subtly foster environmental consciousness among employees. Through this process, employees gradually accept leader’s environmental norms, internalize them into practical actions, and take the initiative to exhibit OCBE. Ethical leaders not only set an example for employees in environmental protection, but also provide resources and guidance for employees to engage in OCBE. As a result, under the influence of ethical leadership, employees gradually identify with leader’s environmental protection values, change their negative attitudes towards environmental protection, and exhibit positive OCBE (Kark and Shamir, 2002). Previous studies have also found that ethical leadership facilitates subordinates’ ethical behavior and employees’ OCBE (Avey et al., 2011; Lu and Lin, 2014). Therefore, we propose the following hypothesis:

*H1*: Individual-level ethical leadership positively influences employees’ OCBE.

### 2.3 Team-level ethical leadership and employees’ OCBE

Team-level ethical leadership refers to the collective perception of leaders’ ethical values, standards, and behaviors among team members (Liao and Chuang, 2007). Ethical leaders, on one hand, adhere to high environmental standards, actively implement the organization’s environmental philosophy, and continuously enhance team members’ environmental awareness. On the other hand, they establish informal rules and norms related to environmental protection within the team by communicating and sharing green values with team members (Trevino et al., 2000; Mayer et al., 2012). Ethical leaders’ environmental behaviors and philosophy serve as positive role models that inspire team members to learn from and emulate ethical leaders’ behaviors, ultimately leading to the display of pro-environmental behaviors. Therefore, we propose the following hypothesis:

*H2*: Team-level ethical leadership positively influences employees’ OCBE.

### 2.4 The mediating effect of leader identity

Existing studies tend to adopt Bandura’s (1977) social learning theory (SLT) as the theoretical lens that shed light on the mechanism underlying the relationship between ethical leadership and employees’ OCBE. For instance, Islam et al. (2021a) posits that employees will socially learn from ethical leaders and hence exhibit more OCBE as they generate a sense of obligation towards the society and the environment by observing ethical leaders’ pro-environmental behavior. However, some scholars countered that such social learning process is dependent on how they identify with the leader (Wang et al., 2021). This argument is also in line with the findings of Ashforth et al. (2016) that employees’ identification with their leader can serve as a medium whereby leadership exerts its impacts on employee altitude and behavior. To this end, we incorporate the identity factors, particularly leader identity, into our research model to examine the mechanism that governs the relationship between ethical leadership and employees’ OCBE. Against this backdrop, we employ social identity theory as a critical facet of the social identity approach to elucidate this mechanism. Social identity theory suggests that individuals will develop a sense of identity and engage in behaviors that benefit the group when important members of an organization or group (e.g., the leader of the team) are able to meet their needs (Ashforth et al., 2008). As previously mentioned, ethical leaders exhibits both moral people and moral managers, specifically, they exhibit high levels of moral standards, actively advocate environmental protection concepts, share environmental values, and demonstrate ethical behaviors such as resource conservation and environmental care. Thus, ethical leaders can benefit both organizational and personal needs. During the frequent interaction with employees, ethical leaders’ environmental values and goals could be gradually aligned with employees and this will in turn generates employees’ identification with the leader. This sense of identification with the leader is formed by employees when they incorporate leaders’ beliefs and values into their self-concept (Kark et al., 2003). The more employees identify with the leader, the more likely they are to internalize ethical leaders’ environmental values and goals as self-behavioral norms and exhibit pro-environmental behaviors similar to the leaders such as OCBE (Zhao and Zhou, 2019). Following this logic, we infer that when ethical leaders value the sustainable development of the organization and uphold high environmental standards, compared to other employees, those who identify with the leader will also emphasize these ideas, and as a result, exhibit increased OCBE in response. This inference is also supported by empirical studies. For instance, scholars have proved that leader identity makes employees follow leaders’ examples by imitating their ethical behaviors and exhibiting similar actions (Zhang and Chen, 2013; Ashforth and Schinoff, 2016). In addition, Wang and Rode (2010) have also found that leader identity reinforces leaders’ motivating effects and drives employees to exhibit more OCBE (Wang and Rode, 2010). Therefore, we propose the following hypothesis:

*H3*: Leader identity mediates the relationship between individual-level ethical leadership and employees’ OCBE.

### 2.5 The cross-level mediating effect of team environmental atmosphere

Team environmental atmosphere refers to the employees’ collective perceptions regarding the environmental norms and policies of the team (Khan et al., 2019; Liu and Yu, 2023). As principal sources of information in the workplace, leaders can influence employees’ shared perceptions of the team context, contributing to the formation of the team atmosphere. Specifically, the formation of a positive team environmental atmosphere also hinges on how leaders articulate their environmental policies (Kuenzi and Schminke, 2009). Since ethical leaders focus on the sustainable development of the organization, they are prone to perceive environmental protection as their moral obligation and implement a series of environmental practices (Khan et al., 2019). They promote the execution of these practices among their subordinates via frequent interaction. During this process, employees are profoundly influenced by the leader’s environmental values, and gaining an understanding of their roles in the organization’s sustainable development. They define themselves in reference to the leader’s environmental philosophy and develop a sense of identification with the leader and. This identification with the leader further generates their identification with the team environmental atmosphere that the leader establishes (Ashforth et al., 2016). Therefore, ethical leadership can shape employees’ collective perceptions regarding the environmental policies and form a positive environmental atmosphere on the team basis (Khan et al., 2019). This inference is in consistent with the findings of Ötken and Cenkci (2012) that ethical leadership exerts a positive impact on the formation of an environmentally friendly team atmosphere.

Moreover, current research indicates that the establishment of an organizational climate significantly influences employee attitudes and behaviors (Tian et al., 2020). As observed by Teresi et al. (2019), the specific culture or climate within diverse organizations greatly affects the way employees behave. The unique organizational climate governs and shapes the actions, decisions, and relationships of individuals within the organization. For instance, ethical climate which places ethical norms at the organization’s core can encourage employees to display more OCB via the process of identification with the organization (Pagliaro et al., 2018). According to this logic, we suggest that positive team environmental atmosphere that values the administrated environmental policies can foster a higher incidence of extra-role green behavior in employees, such as OCBE. Specifically, team environmental atmosphere not only bolsters employees’ environmental awareness but also provides employees with necessary physical resources and psychological support to demonstrate OCBE (Ning and Zhaoyi, 2017). To conclude, ethical leadership shapes team-level environmental atmosphere, and this environmental atmosphere in turn promotes employees to conduct increased OCBE. Therefore, we propose the following hypothesis:

*H4*: Team environmental atmosphere mediates the relationship between team-level ethical leadership and employees’ OCBE.

### 2.6 The cross-level moderating effect of team environmental atmosphere

Prior research has also shown that team environmental atmosphere plays a crucial role in shaping employee behavior and attitudes within organizations (Kozlowski, 2018). Team environmental atmosphere enables employees to understand the team’s environmental values, goals and expectations. Consequently, the team’s ethical standards are internalized as part of the employees’ values, which facilitates their exhibition of OCBE (Lee and Ha-Brookshire, 2018). Moreover, a strong team environmental atmosphere will help employees develop a strong commitment to environmental protection and clarify the team’s environmental goals, which fosters employees’ positive behaviors, including OCBE (Bolino et al., 2004). In a team with a strong environmental atmosphere, employees are aware of the team’s expectation of ethical behaviors such as environmental protection, making them more likely to internalize the team’s environmental values, thus strengthening the influence of individual-level ethical leadership on employees’ OCBE. Conversely, in a team with a weak environmental atmosphere, team members are less influenced by the environmental atmosphere, have a weak commitment to environmental protection, and are limited to fulfilling social obligations, which will reduce the influence of individual-level ethical leadership on employees’ OCBE. Therefore, we propose the following hypothesis:

*H5*: Team environmental atmosphere positively moderates the relationship between individual-level ethical leadership and employees’ OCBE.

**Figure 1:**
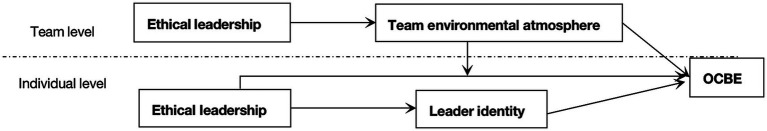
Research model.

Based on the above discussion, our study emphasizes the crucial role that employees plays in addressing environmental issues via their discretionary, ethically-driven behaviors. However, current studies offering insights on how to promote green behaviors at the micro-level of employees in the organizational context are still scant (Ahmad et al., 2021; Islam et al., 2021a). In addition, as noted by Khan et al. (2019), the calls for investigating how leadership contributes to employee pro-environmental behaviors have been growing. In particular, the underlying mechanisms of how ethical leadership affects employees’ OCBE should be explored (Ahmad et al., 2021), especially its multi-level influential mechanism (Norton et al., 2015; Temminck et al., 2015; Luu, 2019). Therefore, our study advances both ethical leadership and OCBE literature by revealing the cross-level influential mechanism of ethical leadership on employees’ OCBE from both an individual and team perspective. In summary, the model of this research is shown in Figure 1.

## 3 Methods

### 3.1 Data collection

The primary purpose of this study was to investigate the cross-level mechanism of ethical leadership on employees’ OCBE. Therefore, the target population of this study was team leaders and their subordinated employees at work from 20 manufacturing companies located in Guangdong Province, South China. Of these 20 companies, three were automotive manufacturers, five were electronic information manufacturers, four were pharmaceutical manufacturers, four were apparel textile manufacturers, and four were oil, gas, electricity, and architecture manufacturers. The choice of this region for our study sample was informed by its standing as one of China’s largest manufacturing bases (Wang et al., 2023). The manufacturing output value in this region contributes to approximately 95% of the whole province’s total industrial output value (Lei et al., 2022). Large-scale agglomeration of the manufacturing industry results in excessive resource consumption and serious environmental pollution. Therefore, compared to other provinces in China, this region are confronted with more environmental challenges. The promotion of employees’ OCBE, as the extension of employee green behaviors, holds greater significance for the sustainable development of this region.

In this study, the convenience sampling method in non-probability sampling was implemented to distribute questionnaires. Prior to the formal survey, we contacted each company’s human resource managers to obtain their consent. Then, we conducted a two-wave distribution of questionnaires to collect data from January to March 2022. In the first wave of survey (in January 2022), leaders’ demographic information and data about employees’ OCBE and team environmental atmosphere were obtained from team leaders. We requested the leaders to provide the respective personnel number of each subordinate they had reported in the questionnaires. Approximately 2 months later, in the second wave of survey (in March 2022), we distributed the employee questionnaires to team leaders’ subordinates via e-mail to obtain their demographics and data on ethical leadership and leader identity. To address any concerns subordinates might have about reporting their direct leader’s ethical behaviors at workplace, we assured them right at the start of the questionnaire that their participation was voluntary and their responses would be kept confidential. Furthermore, we also adopted the reverse scoring method to mitigate the potential bias. We coded all the respondents based on their personnel numbers to match the leader-employee questionnaires collected at two different waves. Finally, a total of 347 employee questionnaires (valid return rate of 90.34%) and 47 leader questionnaires (valid return rate of 87.04%) were obtained after matching every leader-employee questionnaire and eliminating unmatched and incomplete responses. Each team leader evaluated about 7 to 8 subordinates and the average team size was 7.38.

The statistical characteristics of the surveyed employees were as follows: (1) Gender: 47.6% were male and 52.4% were female. (2) Age: 23.1% were aged 25 or younger, 24.2% were aged 26–30, 27.1% were aged 31–40, and 25.6% were aged 41 or older. (3) Educational background: 33.1% held a junior college degree or lower level of education, 36.3% held a bachelor’s degree, and 30.6% held a master’s degree. (4) Tenure: 45.5% had worked for less than 3 years, 40.3% had 4–8 years, 8.4% had 9–14 years, and 5.8% had worked for more than 15 years.

The statistical characteristics of the surveyed team leaders were as follows: (1) Gender: 48.9% were female and 51.1% were male. (2) Age: 40.4% were aged 26–30, 36.2% were aged 31–40, 23.4% were aged 41 or older. (3) Educational background: 6.4% held a junior college degree or lower level of education, 51% held a bachelor’s degree, and 42.6% held a master’s degree. (4) Position: first-line leaders accounted for 57.4% of the sample, middle leaders accounted for 42.6%, and top leaders accounted for 27.6%. (5) Tenure: 27.7% had less than 3 years of work experience, 34% had 4–8 years of work experience, and 38.3% had 9–14 years of work experience.

### 3.2 Variable measurement

In our study, we utilized a five-point Likert Scale in the questionnaire, which ranged from 1 (“strongly disagree”) to 5 (“strongly agree”). Since the adopted measurement scales were originally in English, we first translated them into Chinese. Then, we invited two English professionals to back-translate the scales. After consulting with another two OBHR (Organizational Behavior and Human Resource Management) specialists, we adjusted the expressions in the scales to make them more accurate in Chinese context and finally developed the Chinese version questionnaires.

#### 3.2.1 Individual-level ethical leadership

The 10-item scale proposed by Brown et al. (2005) was used and modified appropriately, with sample items such as “My supervisor always follows environmental norms at work,” and the Cronbach’s alpha coefficient was 0.93.

#### 3.2.2 Team environmental atmosphere

The scale developed by Victor and Cullen (1988) and Mayer et al. (2010) with proper modifications to fit the research on environmental atmosphere, there were overall 9 items, including “The employees in my team maintain higher environmental standards,” etc. The Cronbach’s alpha coefficient was 0.92.

#### 3.2.3 Organizational citizenship behavior for the environment

The 9-item scale of Lamm et al. (2013) was applied, with sample questions such as “My employees turn off the lights whenever leave the office,” and the Cronbach’s alpha coefficient was 0.92.

#### 3.2.4 Leader identity

The 11-item scale of Kark et al. (2003) was adopted, with sample questions such as “I believe that my leader’s success is my success,” and the Cronbach’s alpha coefficient was 0.94.

#### 3.2.5 Control variables

Current studies have found that employees and leaders’ demographic variables such as gender and age exert varying impacts on this research (Farh et al., 2007). In light of this, we controlled employees and leaders’ gender (0 = “male”; 1 = “female”), age (1 = “younger than 25″; 2 = “aged 26–30″; 3 = “aged 31–40″; 4 = “older than 41″), educational background (1 = “junior college degree”; 2 = “bachelor’s degree”; 3 = “master’s degree and above”), position (1 = “first-line leaders”; 2 = “middle leaders”; 3 = “top leaders”), and tenure (1 = “less than 3 years”; 2 = “4–8 years”; 3 = “9–14 years”; 4 = “more than 15 years”) in this study.

### 3.3 Statistical method

This study used SPSS 24.0, AMOS 23.0, and HLM 6.02 to conduct descriptive statistical analysis, confirmatory factor analysis, and hypothesis test. Before data analysis was conducted, this study primarily determines whether individual-level data can be aggregated at the team level by calculating the inter-rater agreement (*R*_wg_) and the intra-class correlations (ICCs) for team-level ethical leadership and team environmental atmosphere. The results showed that the mean values of *R*_wg_ for team-level ethical leadership and team environmental atmosphere were 0.76 and 0.91, respectively, both exceeding the critical value of 0.70 (Bliese, 2000), indicating consistency in employee perceptions of ethical leadership and team environmental atmosphere within the same team and thus justifying the aggregation of data. Furthermore, the team-level ethical leadership ICC1 was 0.44, and ICC2 was 0.85; the team environmental atmosphere ICC1 was 0.41, and ICC2 was 0.84. Both ICC1 values for these two variables were greater than 0.10. The chi-square test showed significant inter-group variances, indicating a significant difference in team-level ethical leadership and team environmental atmosphere. Both ICC2 values for these two variables were greater than 0.7, indicating good reliability of team-level variables (James, 1982; James et al., 1984).

## 4 Empirical analysis

### 4.1 Common method variance

As previously presented, to mitigate the potential impact of common method variance (CMV), we collected data in two waves from dual sources: employees and their direct leaders (Podsakoff et al., 2012). Despite this, the cross-sectional nature of the data could still potentially incur CMV. To address this, we conducted Harman’s single-factor test and confirmatory factor analysis. The results of Harman’s single-factor test showed that under principal component analysis, the first factor explained only 32.7% of the variation, falling below the Harman’s recommended 40% standard (Harman, 1976), and even well below the threshold value of 50% (Podsakoff et al., 2003). In addition, we carried out confirmatory factor analysis using AMOS 23.0 and the results (Table 1) showed that the single-factor model, in which all items were loaded on the single common factor, demonstrated a significantly inferior fit in the data (χ^2^/df = 7.84, RMSEA = 0.143, CFI = 0.46, TLI = 0.43). Therefore, these results indicate that CMV was not a serious issue in this study.

**Table 1:** Confirmatory factor analysis.

Model	χ^2^	df	χ^2^/df	RMSEA	CFI	TLI	SRMR
Model 1: EL, TE, LI, EB	838.10	696	1.20	0.033	0.98	0.98	0.03
Model 2: EL, TE, LI + EB	2146.64	699	3.07	0.080	0.84	0.83	0.09
Model 3: EL, TE + LI + EB	4014.79	701	5.72	0.116	0.63	0.61	0.15
Model 4: EL + TE + LI + EB	5503.97	702	7.84	0.143	0.46	0.43	0.17

EL refers to individual-level ethical leadership; TE refers to team environmental atmosphere; LI refers to leader identity; EB refers to employees’ OCBE.

### 4.2 Discriminant validity test

In this study, confirmatory factor analysis was also conducted to test the discriminant validity and convergent validity (CV) of four variables: individual-level ethical leadership, team environmental atmosphere, leader identity, and employees’ OCBE. As shown in Table 1, the four-factor model had the best fit (χ^2^/df = 1.20, RMSEA = 0.033, CFI = 0.98, TLI = 0.98), indicating ideal discriminant validity among the four variables.

Table 2 presents the assessment of convergent validity of each variable. First, factor loadings range from 0.69 to 0.88, which is in the acceptable range (above 0.50). Second, the CR values of ethical leadership, team environmental atmosphere, employees’ OCBE, and leader identity are 0.93, 0.93, 0.92, and 0.94, respectively, which are all greater than the 0.70 benchmark. Last, the AVE values are 0.59, 0.59, 0.57, and 0.60, respectively, which also exceed the 0.50 critical value. These results have further confirmed the validity of each variable.

**Table 2:** Convergent validity of each variable.

Variable	Items	Factor loadings	CR	AVE
EL	EL1	0.78	0.93	0.59
	EL2	0.82		
	EL3	0.74		
	EL4	0.70		
	EL5	0.75		
	EL6	0.72		
	EL7	0.75		
	EL8	0.74		
	EL9	0.88		
	EL10	0.77		
TE	TE1	0.84	0.93	0.59
	TE2	0.78		
	TE3	0.77		
	TE4	0.66		
	TE5	0.74		
	TE6	0.75		
	TE7	0.72		
	TE8	0.80		
	TE9	0.82		
EB	EB1	0.70	0.92	0.57
	EB2	0.80		
	EB3	0.81		
	EB4	0.77		
	EB5	0.73		
	EB6	0.73		
	EB7	0.86		
	EB8	0.70		
	EB9	0.71		
LI	LI1	0.83	0.94	0.60
	LI2	0.84		
	LI3	0.73		
	LI4	0.79		
	LI5	0.69		
	LI6	0.77		
	LI7	0.78		
	LI8	0.82		
	LI9	0.78		
	LI10	0.78		
	LI11	0.71		

EL refers to individual-level ethical leadership; TE refers to team environmental atmosphere; LI refers to leader identity; EB refers to employees’ OCBE.

### 4.3 Descriptive statistical analysis

Table 3 shows the means, standard deviations, and correlation coefficients of the variables. From Table 3, at the individual level, ethical leadership is positively correlated with employees’ OCBE (*r* = 0.41, *p* < 0.001) and leader identity (*r* = 0.46, *p* < 0.001), and leader identity is positively correlated with employees’ OCBE (*r* = 0.50, *p* < 0.001). At the team level, ethical leadership is positively correlated with team environmental atmosphere (*r* = 0.54, *p* < 0.001), and these results have provided a good basis for the rest of this study.

**Table 3:** The means, standard deviations, and correlation coefficient of the variables.

Variables	*M*	SD	1	2	3	4	5	6	7
**Individual-level variables** (*N* = 347)									
1. Employee’s gender	0.52	0.50	1						
2. Employee’s age	2.55	1.10	0.02	1					
3. Employee’s education background	1.97	0.79	0.04	0.15^**^	1				
4. Employee’s tenure	1.74	0.84	−0.09	−0.05	−0.10	1			
5. Ethical leadership	3.49	0.98	−0.04	−0.05	−0.04	0.14^**^	1		
6. Leader identity	3.32	0.97	0.03	−0.02	−0.01	0.01	0.46^***^	1	
7. OCBE	3.54	0.87	−0.01	0.01	0.11	0.19^***^	0.41^***^	0.50^***^	1
**Team-level variables** (*N* = 47)									
1. Leader’s gender	1.48	0.50	1						
2. Leader’s age	2.83	0.78	0.10	1					
3. Leader’s education background	2.36	0.60	0.19	−0.09	1				
4. Leader’s position	1.97	0.76	0.36^*^	0.06	0.20	1			
5. Leader’s tenure	2.10	0.81	0.13	−0.10	0.01	−0.20	1		
6. Ethical leadership	3.49	0.70	0.17	−0.07	0.09	−0.13	0.34^***^	1	
7. Team environmental atmosphere	3.55	0.54	0.09	−0.07	0.03	−0.02	0.34^*^	0.54^***^	1

*p < 0.05, **p < 0.01, and ***p < 0.001.

### 4.4 Hypothesis test

#### 4.4.1 The relationship between individual-level ethical leadership and employees’ OCBE

Before conducting hypothesis analysis, a null model was established with employees’ OCBE as the dependent variable to test whether there existed significant inter-group variance. The results showed that τ = 0.37, σ^2^ = 0.38, ICC1 = 0.49, and the chi-square test for inter-group variance was significant (χ^2^ = 374.91, *p* < 0.001) with a degree of freedom of 46. This indicated that 49% of the variance in the outcome variable came from inter-group variance, which satisfied the conditions for cross-level analysis.

As shown in Table 4, at the individual level, ethical leadership had a significant positive effect on employees’ OCBE (M3, *β* = 0.29, *p* < 0.001). Thus Hypothesis 1 was supported. The test for mediating effects in this study was based on the four-step approach proposed by Kenny et al. (2003). In the first step, ethical leadership had a significant positive effect on employees’ OCBE (Hypothesis 1 supported). In the second step, ethical leadership had a significant positive effect on leader identity (M2, *β* = 0.43, *p* < 0.001). In the third step, leader identity had a significant positive effect on employees’ OCBE (M4, *β* = 0.45, *p* < 0.001). Finally, when ethical leadership and leader identity were both included in the model, the results showed that there was a significant positive correlation between ethical leadership and employees’ OCBE, which indicates that leader identity partially mediated the relationship between individual-level ethical leadership and employees’ OCBE (M5, *β* = 0.13, *p* < 0.01). The results of the bootstrap method showed that the indirect effect value of leader identity between ethical leadership and employees’ OCBE was 0.165, with a confidence interval of [0.115, 0.220], which excluded 0. These results supported Hypothesis 3.

**Table 4:** The results of HLM analysis of ethical leadership and OCBE.

Variables	Leader identity		OCBE			
	M1	M2	M3	M4	M5	M6
Intercept term	3.33^***^	3.33^***^	3.55^***^	3.55^***^	3.54^***^	3.54^***^
**Individual level**						
Employee’s gender	−0.01	−0.01	−0.08	−0.07	−0.08	−0.06
Employee’s age	0.00	0.02	0.02	0.01	0.02	0.02
Employee’s education background	0.02	0.04	0.09^**^	0.07	0.07^*^	0.09^**^
Employee’s tenure	−0.05	−0.07^*^	0.15^***^	0.19^***^	0.18^***^	0.16^***^
Individual-level ethical leadership		0.43^***^	0.29^***^		0.13^**^	0.30^***^
Leader identity				0.45^***^	0.39^***^	
Ethical leadership × team environmental atmosphere						0.26^**^
**Team level**						
			**M7**	**M8**	**M9**	**M10**
Leader’s gender			0.10	−0.06	−0.03	−0.06
Leader’s age			0.14	0.20^*^	0.22^*^	0.27^*^
Leader’s education background			0.07	−0.11	−0.07	−0.06
Leader’s position			0.02	0.09	0.06	0.13
Leader’s tenure			0.30^*^	−0.06	0.14	−0.03
Team-level ethical leadership				0.54^**^		0.36
Team environmental atmosphere					0.55^**^	0.50^**^
Intra-group variance			0.39	0.32	0.26	0.25
Inter-group variance			0.48	0.29	0.28	0.27
Model variance			793.73	712.75	655.40	646.89

*p < 0.05, **p < 0.01, and ***p < 0.001.

#### 4.4.2 The relationship between team-level ethical leadership and employees’ OCBE

The results in Table 4 showed that team-level ethical leadership had a significantly positive impact on employees’ OCBE (M8, *β* = 0.54, *p* < 0.01). Thus, Hypothesis 2 was supported. Cross-level mediating effects were also tested according to the four-step approach. The results from M8-M12 (Tables 4, 5) showed that the relationship between team-level ethical leadership and employees’ OCBE was completely mediated by team environmental atmosphere (M10, *β* = 0.36, *p* < 0.01). Therefore, Hypothesis 4 was supported.

**Table 5:** The OLS regression analysis of team-level ethical leadership and team environmental atmosphere.

Variables	Team environmental atmosphere	
	M11	M12
Leader’s gender	0.036	−0.012
Leader’s age	−0.032	−0.034
Leader’s education background	0.012	−0.033
Leader’s position	0.024	0.035
Leader’s tenure	0.230^*^	−0.083
Team-level ethical leadership		0.491^**^
*R^2^*	0.127	0.306
*△R^2^*	0.127	0,179
*F*	1.197	2.943^**^
*△F*	1.197	10.313

*p < 0.05, **p < 0.01, and ***p < 0.001.

#### 4.4.3 The cross-level moderating effect of team environmental atmosphere

**Figure 2:**
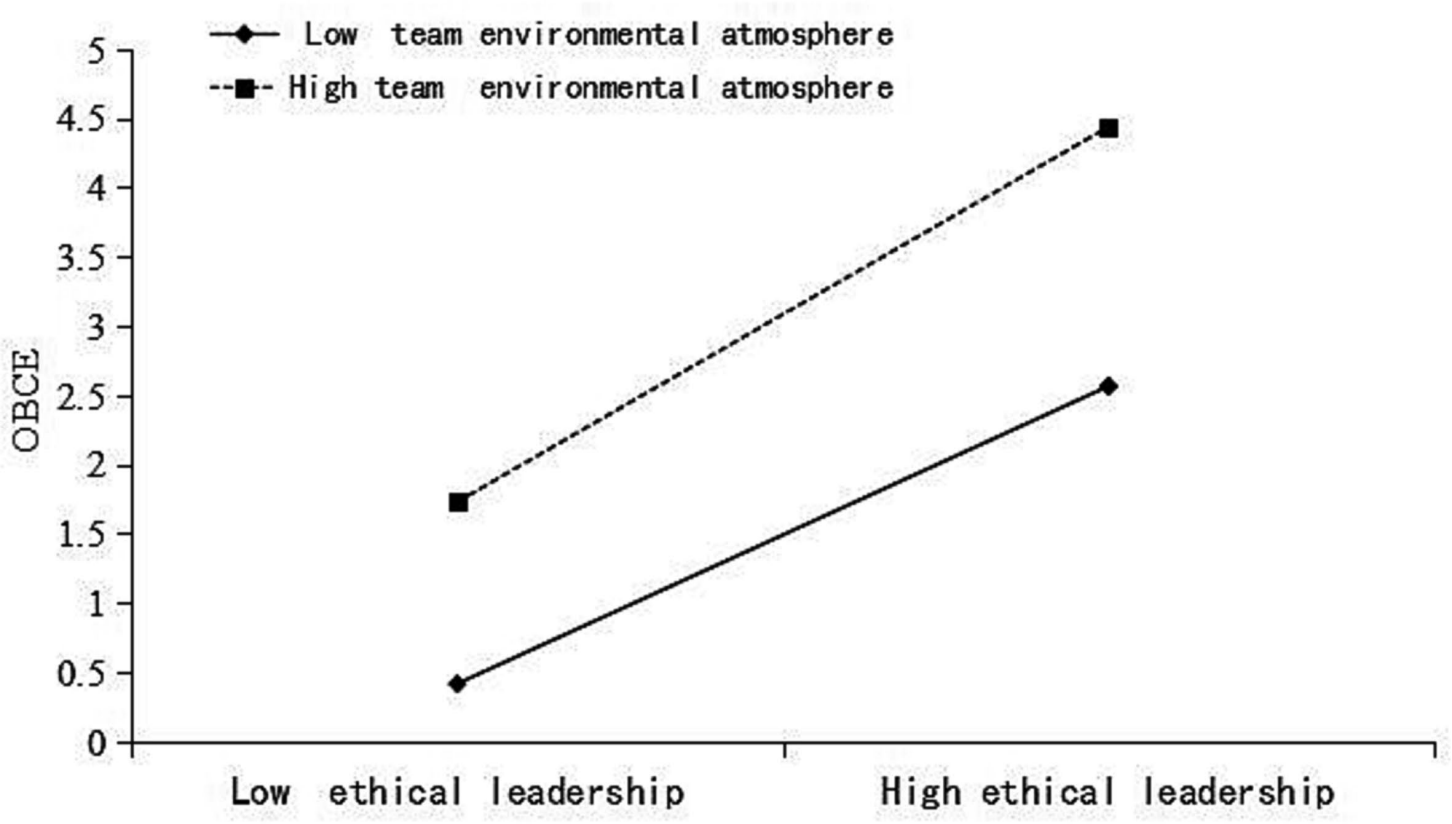
Moderating effect of team environmental atmosphere on the relationship between ethical leadership and OCBE.

The empirical results of M6 in Table 4 showed that the interaction term between team environmental atmosphere and individual-level ethical leadership had a significant positive effect on employees’ OCBE (M6, *β* = 0.26, *p* < 0.01), and the results of the slope analysis showed that, compared to weaker team environmental atmosphere, ethical leadership had a stronger impact on employees’ OCBE when the team environmental atmosphere was stronger (the moderating effect is shown in Figure 2). Therefore, team environmental atmosphere had a cross-level moderating effect on the relationship between ethical leadership and employees’ OCBE, and hence Hypothesis 5 was supported.

## 5 Conclusion and discussion

### 5.1 Research findings

Based on social identity theory, this study constructed a cross-level theoretical model of ethical leadership on employees’ OCBE and empirically tested this model by taking 347 pairs of leader-employee questionnaires as a sample. The findings reveal the followings: (1) individual-level ethical leadership contributes to employees’ OCBE; (2) team-level ethical leadership also has a positive impact on employees’ OCBE; (3) leader identity partially mediates the relationship between individual-level ethical leadership and employees’ OCBE; (4) team environmental atmosphere completely mediates the relationship between team-level ethical leadership and employees’ OCBE; (5) team environmental atmosphere moderates the relationship between individual-level ethical leadership and employees’ OCBE across levels.

### 5.2 Theoretical contribution

First, existing literature on corporate environmental protection has been mainly conducted at the organizational macro level, such as corporate strategy and operations (Galpin and Whittington, 2012), while micro-level studies on environmental protection behavior from the employees’ perspective are a new research field that has emerged in recent years. In fact, recent studies have indicated the crucial role employees plays in addressing the ethical and environmental issues via their extra-role behavior, especially OCBE (Zhao and Zhou, 2019; Ahmad et al., 2021; Islam et al., 2021b; Liu and Yu, 2023). Although employees’ OCBE is hardly regulated by organizations, it can effectively compensate for the lack of formal rules (Lamm et al., 2013; Cheema et al., 2019). Employees’ OCBE can improve the environmental performance and accelerate companies’ green transformation and sustainable development (Ahmad et al., 2021; Islam et al., 2021a,b; Liu and Yu, 2023). However, there is a dearth of studies offering insights on how to promote green behaviors at the micro-level of employees in the organizational context (Ahmad et al., 2021). Therefore, the exploration of ethical leadership’s influence on employees’ OCBE in this study enriches the micro-level research on corporate environmental behavior, which will be helpful in filling the gaps in the micro-level research literature.

Second, existing literature on the relationship between ethical leadership and employees’ OCBE mainly focused on the individual level, yet paid less attention to the team level, which cannot fully explain the influence mechanism of ethical leadership on employees’ OCBE. In addition, existing research has rarely explored the influence of ethical leadership on employees’ OCBE at both the individual and team levels, and there is a lack of research on the cross-level influence mechanism of ethical leadership in the team context (Luu, 2019). However, this study provides an in-depth analysis of the cross-level impact of multi-level ethical leadership on employees’ OCBE from both individual level and team level, responding to the call by scholars such as Norton et al. (2015) and Temminck et al. (2015) for more comprehensive research on leadership. The findings of this study indicate that both individual-level ethical leadership and team-level ethical leadership have a positive impact on employees’ OCBE. However, research solely focused on the individual level cannot fully demonstrate the impact of ethical leadership on employees’ OCBE. Furthermore, as a leadership style characterized by Chinese Confucianism, ethical leadership focuses on the harmonious development of the relationship between organizational sustainability and environmental protection, and the empirical study on ethical leadership’s influence on employees’ OCBE in this study is helpful in deepening the understanding of ethical leadership with Chinese cultural characteristics.

Third, existing literature on the influence mechanisms of ethical leadership on employees’ OCBE mainly adopted the affective event theory, social information theory, and social learning theory (Robertson and Barling, 2013; Khan et al., 2019; Islam et al., 2021a). Despite these theories examining the impact of individual identity and organizational identity on the relationship between leadership style and employee attitudes and behaviors, the consideration of leader identity has been relatively overlooked (Zhao and Zhou, 2019). In addition, whether subordinates socially learn from their leaders is also dependent on how they identify with their leaders (Wang et al., 2021). That said, leader identity serves as a link that bridges leadership and employee behavior (Ashforth et al., 2016), and hence it is of great necessity to discuss the role of leader identity. Based on social identity theory, this study incorporated leader identity in the research model to analyze the cross-level influence mechanism of ethical leadership on employees’ OCBE and reveals the “black box” of the relationship between ethical leadership and employees’ OCBE in a more comprehensive way. The results show that leader identity partly mediates the relationship between individual-level ethical leadership and employees’ OCBE, team environmental atmosphere completely mediates the relationship between team-level ethical leadership and employees’ OCBE, and team environmental atmosphere positively moderates the relationship between individual-level ethical leadership and employees’ OCBE across levels. This study extends our understandings of the leadership’s influence on both subordinates’ identification and their behavior (Ishaq et al., 2023). These findings in this study also respond to the call by Khan et al. (2019) for more studies on the mediating mechanism between ethical leadership and employees’ OCBE.

### 5.3 Managerial implications

Our research points the way for managers to receive positive responses and support from employees in environmental protection. First, ethical leaders possess good moral qualities and set a good example of environmental protection, which can encourage employees to take the initiative to demonstrate OCBE. In light of this, team leaders should develop their ethical leadership. Specifically, team leaders are supposed to focus on improving their own environmental standards, paying attention to the sustainable development and environmental issues of the organization, and setting an example for employees.

Second, the demonstration of employees’ OCBE is inseparable from the team context. In the context of a positive team environmental atmosphere, employees would focus on incorporating their personal values with team’s environmental values, which can contribute to more employees’ OCBE. Based on this, team leaders should establish a positive team environmental atmosphere by emphasizing pro-environmental values, providing employees with emotional support and necessary resources. In addition, the implementation of team-based rewards that focus on environmental standards would also be helpful to foster a pro-environmental atmosphere.

Third, leader identity serves as a crucial connector between ethical leadership and employees’ OCBE. On account of this, team leaders should generate employees’ identification through regular interactions. On the one hand, team leaders should adhere to elevated environmental standards, thus modeling ideal behavior for employees; on the other hand, it is also important for team leaders to impart their environmental values to employees (Luu, 2019).

### 5.4 Limitations and future research

While these findings provide valuable insights into the cross-level relationship between ethical leadership and employees’ OCBE, it is necessary to acknowledge certain limitations of this study. First, despite adopting several strategies to mitigate the impact of common method variance, such as utilizing matched sample data and two-phase data collection (Liu and Yu, 2023), our research still predominantly relies on cross-sectional data. Consequently, we highly recommend future research to undertake longitudinal studies where data is gathered at different time intervals, to provide a more rigorous validation of the cross-level model.

In addition, OCBE described in this study mainly pertains to individual-level behavior of employees. However, the complex nature of employee OCBE indicates that the interaction among employees with teams could also shape team-level OCBE (Khan et al., 2019; Liu and Yu, 2023). Therefore, future studies can consider exploring the influential mechanism of team-level ethical leadership on team-level OCBE to further enrich the research on multilevel OCBE.

Furthermore, this study was conducted using a sample primarily composed of manufacturing companies, and hence we recommend that future studies consider incorporating service industry companies into their sample to obtain a more comprehensive understanding of the phenomenon. For example, Khan et al. (2019) collected data from a broad range of manufacturing and service sector companies in China to investigate the impact of ethical leadership on employee OCBE.

## Data availability statement

The raw data supporting the conclusions of this article will be made available by the authors, without undue reservation.

## Ethics statement

Ethical review and approval was not required for the study on human participants in accordance with the local legislation and institutional requirements. Written informed consent from the patients/participants or patients/participants legal guardian/next of kin was not required to participate in this study in accordance with the national legislation and the institutional requirements.

## Author contributions

XS: Conceptualization, Funding acquisition, Methodology, Resources, Supervision, Writing – original draft, Writing – review & editing, Investigation. HW: Conceptualization, Supervision, Writing – original draft, Writing – review & editing, Investigation. YZ: Conceptualization, Software, Writing – original draft, Writing – review & editing, Investigation, Methodology.
